# Detection of Giardia lamblia by Microscopic Examination, Rapid Chromatographic Immunoassay Test, and Molecular Technique

**DOI:** 10.7759/cureus.10287

**Published:** 2020-09-07

**Authors:** Amjad Alharbi, Fawzia H Toulah, Majed H Wakid, Esam Azhar, Suha Farraj, Ahmed A Mirza

**Affiliations:** 1 Faculty of Science for Girls, Department of Biology, King Abdulaziz University, Jeddah, SAU; 2 College of Science, Department of Biology, University of Jeddah, Jeddah, SAU; 3 Faculty of Applied Medical Sciences, Department of Medical Laboratory Sciences, King Abdulaziz University, Jeddah, SAU; 4 Special Infectious Agents Unit, King Fahd Medical Research Center, Jeddah, SAU

**Keywords:** giardia lamblia, diagnosis, parasites, real-time pcr, neglected diseases, saudi arabia, icts

## Abstract

Background

*Giardia lamblia* is a pathogenic intestinal flagellate transmitted by the ingestion of contaminated water or food with the cyst stage of the parasite. Giardiasis can cause severe acute diarrhea and malabsorption or may persist as a chronic infection. Effective treatment and control measures depend on proper laboratory diagnosis using diagnostic methods with high sensitivity and specificity.

Objective

To compare the sensitivity and specificity of direct smear, Ritchie sedimentation technique, two brands of rapid chromatographic immunoassay test, and real-time polymerase chain reaction (PCR) for the detection of *G. lamblia* in clinical human fecal samples.

Materials and methods

Unpreserved 100 stool specimens were collected in clean plastic containers and labeled with the patient’s information and examined through light microscopy, immunochromatographic test (ICTs), and real-time PCR.

Results

Out of 100 fresh stool samples obtained from workers analyzed, real-time PCR targeting the SSU rRNA gene was able to detect *Giardia *deoxyribonucleic acid (DNA) in (42) samples followed by ImmunoCard STAT! (31) samples (Meridian Bioscience, Germany), direct smear (23) samples, CerTest (19) samples (Biotec, Zaragoza, Spain), and Ritchie technique (17) samples. Real-time PCR was the most sensitive for the diagnosis of *G. lamblia* in comparison to the other techniques.

Conclusions

All the techniques investigated were sensitive for the detection of *G. lamblia* in stool samples. Further studies are recommended using multiplex real-time PCR assay in order to increase the possibility of the presence or absence of the infection.

## Introduction

One of the most important causes of parasitic diseases is a flagellated protozoan called *Giardia lamblia*. The life cycle is simple and direct, including the motile trophozoite and nonmotile cyst stages while the ingestion of cysts in contaminated water or food (fecal-oral route) is the main mode of transmission [1]. The World Health Organization (WHO) reported that 748 million people drink unsafe water, 2.5 billion are without improved sanitation, more than 200 million cases are reported annually, and since 2004, *Giardia* has been included in the "neglected diseases initiative" [2-3]. Infection with *Giardia *in many people is asymptomatic while others suffer from acute watery diarrhea, abdominal cramps, nausea, epigastric pain, malabsorption, flatulence, and weight loss [4]. In some patients and in relation to several factors, the infection may sustain and become chronic [5]. Previous studies in Saudi Arabia reported high prevalence rates of intestinal parasitic diseases with a different rate of *G. lamblia* (2%-20%) among various populations [6-9].

The early accurate diagnosis of giardiasis is important for the successful treatment and prevention of diseases. The routine laboratory diagnosis is performed for the detection of trophozoites or cysts by microscopic examination of at least three stool samples collected independently [10-11]. This diagnosis depends upon the times of sampling, patient compliance, application of concentration methods, and the expertise of the technologists. Furthermore, in recent years, other methods, such as rapid immunochromatographic tests (ICTs) and molecular techniques, are used mainly for diagnostic or research proposes. The sensitivity and specificity of all diagnostic methods can be improved by including alternative diagnostic procedures that are more rapid and reliable [12]. Isolates of* Giardia* are classified into eight genotypes and the studies of these genes have indicated evidence of cryptic infection, suggesting the use of molecular diagnostics [13].

The aim of the present study was to compare the sensitivity and specificity of several diagnostic methods: direct smears, Ritchie sedimentation technique, two brands of ICTs, and real-time PCR for the detection of *G. lamblia* in clinical human fecal samples.

## Materials and methods

Specimen collection

Stool specimens were collected in clean plastic containers from workers recently arrived in Jeddah, Saudi Arabia, during their screening visit to get a residence permit (officially known as Iqama).

Questionnaire form

The data of patients were collected by interviews in a standard questionnaire that was designed for this study. It included questions on demographic information (age, nationality, the period of residence in Saudi Arabia, and occupation) and some general information about intestinal parasitic infections.

Laboratory examination

Laboratory microscopic examination of feces was accomplished by using direct smears, the Ritchie technique, Para-Pak trichrome stain. Two brands of rapid chromatographic immunoassay tests were performed according to the manufacturer's instructions. ImmunoCard STAT! CGE (Meridian, Bioscience, Germany) and CerTest (Biotec, Zaragoza, Spain) are designed to identify *G. lamblia, Cryptosporidium parvum,* and *Entamoeba histolytica*. Real-time PCR was used to detect the small subunit ribosomal RNA gene (SSU rRNA) of *G. lamblia*.

Direct smears

About 2 mg for each stool sample was mixed with a drop of normal saline (0.9%) and iodine on a clean glass slide (75 X 26 mm). A cover glass (22 X 22 mm) was placed carefully and then examined by light microscopy using (10x) and (40x) objective lenses.

Ritchie (formal ether) sedimentation technique

About 2 gm of each stool sample was emulsified in 10 ml of 10% formal-saline. It was then filtered through two gauze layers fitted in a funnel and centrifuged for 5 min at 2000 rpm. The supernatant was discarded, and the sediment was re-suspended in 10 ml of formal saline (10%) and mixed very well. After that, 3 ml of diethyl ether was added, shaken vigorously for 10 sec, and then centrifuged for 5 min at 2000 rpm. Four layers were formed: the top layer of ether, the plug of the debris, 10% formal saline, and the sediment layer at the bottom of the tube. The top three layers were removed, and a drop of iodine was mixed with the sediment and then examined using (10x) and (40x) objective lenses [14].

Para-Pak trichrome stain

Stool samples were fixed in polyvinyl alcohol (PVA). Stool smears were prepared and stained by Wheatley trichrome stain according to the manufacturer's instructions, (Para-Pak® Trichrome Stain, Meridian, Bioscience, Germany). Smears were air-dried then stained with trichrome stain for 6-8 min, placed in acid ethanol for 5-10 sec, then two quick dips in (95%) ethanol. The slides were immersed two times in (95%) ethanol for 5 min, then in (96-100%) ethanol for 3 min, and finally placed in xylene for 3 min. Finally, each slide was mounted with a coverslip (22 X 40 mm) and examined using an oil immersion (100x) objective lens.

ImmunoCard STAT! CGE

For each specimen, 1 ml of the diluent was transferred into a 15 ml centrifuge tube. Thereafter, approximately 50 mg of each stool specimen was added and mixed gently, then centrifuged for 5 min at 3000 rpm. Next, 125 µl of each prepared specimen was placed into the sample port of the device and the results were interpreted after 10 min. A purple band appears at the control line (C), indicating that the test was completed successfully. The blue band at position 1 on the device frame is indicative of the presence of *C. parvum* antigens. In the presence of *G. lamblia* antigens, a red/pink color is observed at band 2. A green band appearing at position 3 is related to *E. histolytica* antigens.

CerTest

One-hundred twenty-five (125) mg was added into the diluent collection tube, mixed very well, and then shaken vigorously for seconds. Four drops of the prepared specimen were dispensed in each of the circular windows. The test was interpreted after 10 min with a green band-of-control line. The formation of a red band at the test line is indicative of the presence of the investigated parasite.

DNA isolation

Stool samples were subjected to DNA extraction using the QIAamp DNA Stool Mini Kit (Qiagen, Germany) protocol. Each DNA sample was eluted in a final volume of 100 μl and stored at -80°C until needed.

Real-time PCR amplification

The target gene of the real-time PCR assay is the SSU rRNA gene. DNA was ampliﬁed and detected for* G. lamblia* speciﬁcally. To select the speciﬁc *Giardia *sequence, alignments were made of 31 GenBank sequences that included 16 *Giardia *and 15 sequences of other parasites and bacteria (non-pathogenic). The *Giardia-*speciﬁc primers and probe set consisted of the forward primer *Giardia *F, the reverse primer *Giardia *R, and the *Giardia *specific double-labeled probe *Giardia *T (Humanizing Genomic Macrogen Clinical Lab, Korea) [15]: *Giardia *F (5՜-GACGGCTCAGGACAACGGTT-3՜; positions 80-99), *Giardia *R (5՜- TTGCCAGCGGTGTCCG-3՜; positions 142-127) and *Giardia *T (FAM-5՜-CCCGCGGCGGTCCCTGCTAG-3՜-TAMRA; positions 105-124).

Stock primers and probe

According to the manufacturer's instructions, to reach 100 pmol/µl, 250 µl of nuclease-free water was added for each primer and 280 µl was added for the probe.

Working primers and probe

Fifty µl from each stock primer was added to 50 µl nuclease-free water to prepare 50 pmol/µl. From this, 10 µl was mixed with 90 µl of nuclease-free water. Thirty µl from the stock probe was added to 70 µl nuclease-free water to prepare 30 pmol/µl. From this, 10 µl was mixed with 90 µl of nuclease-free water. All working primers and probes were stored at -20°C until needed.

 

Real-time PCR master mix preparation

The real-time PCR master mix was prepared according to QuantiTect® Probe PCR Kit instructions (Qiagen, Germany) in a 1.5 ml microcentrifuge tube, vortexed very well, and then briefly centrifuged at full speed. After that, the reaction mix was dispensed in a volume of 15.5 µl using a standard PCR plate containing 96 wells strips tubes. Then, 9.5 µl of the template DNA was added to the individual wells containing the reaction mix. The plate was then transferred into Applied Biosystems 7500 Fast cycler (Foster City, California), to start the cycling program.

Real-time PCR data analysis

Data analysis was performed with the Applied Biosystems 7500 Fast System by calculating the threshold cycle number (Ct value) that presented the positive amplification of gene in a real-time cycler number.

Statistical analysis

Categorical data were reported as frequency and percentage (%). Data were analyzed using SPSS (version 21, IBM Corp., Armonk, NY). A P-value of < 0.05 value was accepted as statistically significant (corresponding to 95% confidence intervals (CI)). Sensitivity, specificity, PPV, NPV, and accuracy were calculated to test the diagnostic yield.

## Results

Out of 100 fecal samples analyzed, 48 were positive for *G. lamblia*, 18 samples for *E. histolytica*, nine samples for *C. parvum*, three samples for *E. coli,* three samples for hookworms, two samples for *Trichuris trichiura*, two samples for *Enterobius vermicularis,* and one sample for each of the following: *Endolimax nana*, *Hymenolepis nana,* and *Taenia* spp.

Table 1 summarizes the demographic characteristics of the study subjects. All subjects were male workers aged from 20 to 53 years (32.09 ± 8.598), mainly from Pakistan. Most of the stool samples were soft (49%) and loose (48%) while three patients produced watery stool.

**Table 1:** Demographic characteristics a: within the same nationality; b: within all infected cases (62)

Country of origin	Frequencies	Infected cases		
	N	n	(%)^a^	(%)^b^
Bangladesh	7	3	42.86	4.84
Egypt	12	6	50	9.68
India	31	18	58.06	29.03
Nepal	2	2	100	3.23
Pakistan	36	27	75	43.55
Philippines	4	1	25	1.61
Sri Lanka	2	1	50	1.61
Sudan	4	3	75	4.84
Yemen	2	1	50	1.61
Total	100	62	---	100

Out of the 100 samples screened for *G. lamblia*, real-time PCR was able to detect *Giardia* DNA in 42 samples followed by ImmunoCard STAT! CGE (31), direct smears (23), CerTest (19), and the Ritchie sedimentation technique (17).

**Figure 1:**
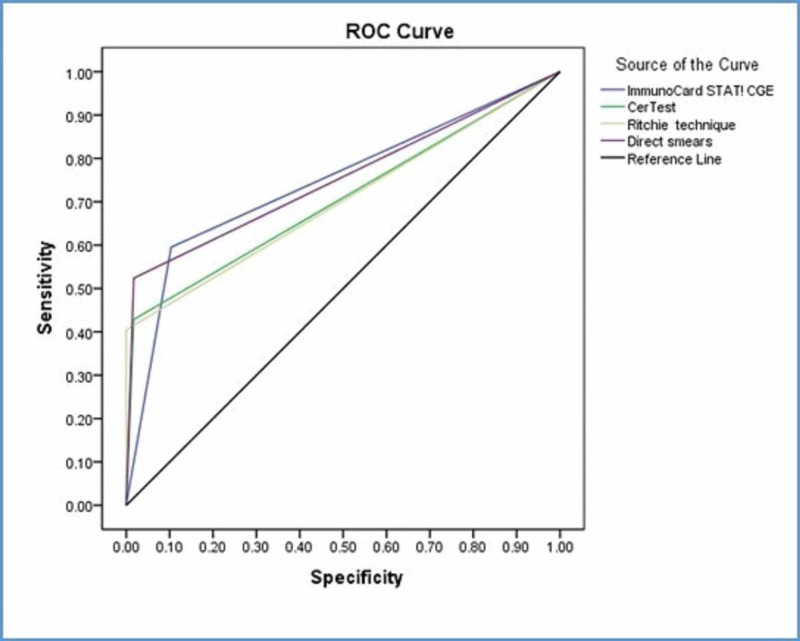
Sensitivity, specificity, and accuracy of diagnosis tools with real-time PCR PCR: polymerase chain reaction

Considering real-time PCR as a nominated golden standard, Immuno*Card* STAT! CGE revealed the highest sensitivity followed by direct smears (Table 2). ImmunoCard STAT! CGE was acceptable. The area under the curve is 0.746, Std. Error=0.053, p-value<0.0005 with 95% CI (0.643-0.849). CerTest was acceptable. The area under the curve is 0.706, Std. Error=0.056, p-value<0.0005 with 95% CI (0.596-0.815). The Ritchie technique was acceptable. The area under the curve is 0.702, Std. Error=0.056, p-value=0.001 with 95% CI (0.592-0.812). Direct smears were acceptable. The area under the curve is 0.753, Std. Error=0.053, p-value<0.0005 with 95% CI (0.649-0.857) (Figure 1).

**Table 2:** Sensitivity, specificity, and accuracy of diagnostic tools with real-time PCR N: Negative, P: Positive, TP: True-positive, FP: False-negative, TN: True-negative, FN: False-negative, PPV: Positive predictive value, NPV: Negative predictive value ImmunoCard STAT!: Meridian Bioscience, Germany; CerTest: Biotec, Zaragoza, Spain

Test		Real-time PCR		Sensitivity %	Specificity %	PPV %	NPV %	95% CI	P-value
		P	N						
Immuno Card STAT! CGE	P	^TP^25	^FP^6	(59.52)	(89.65)	(80.64)	(75.36)	(0.64-0.85)	P < 0.001
	N	^FN^17	^TN^52						
CerTest	P	^TP^18	^FP^1	(42.86)	(98.27)	(94.74)	(70.37)	(0.60-0.82)	P < 0.001
	N	^FN^24	^TN^57						
Ritchie	P	^TP^17	^FP^0	(40.47)	(100)	(100)	(69.88)	(0.60-0.81)	P < 0.001
	N	^FN^25	^TN^58						
Direct smears	P	^TP^22	^FP^1	(52.38)	(98.27)	(95.65)	(74.02)	(0.65-0.86)	P < 0.001
	N	^FN^20	^TN^57						

**Figure 2:**
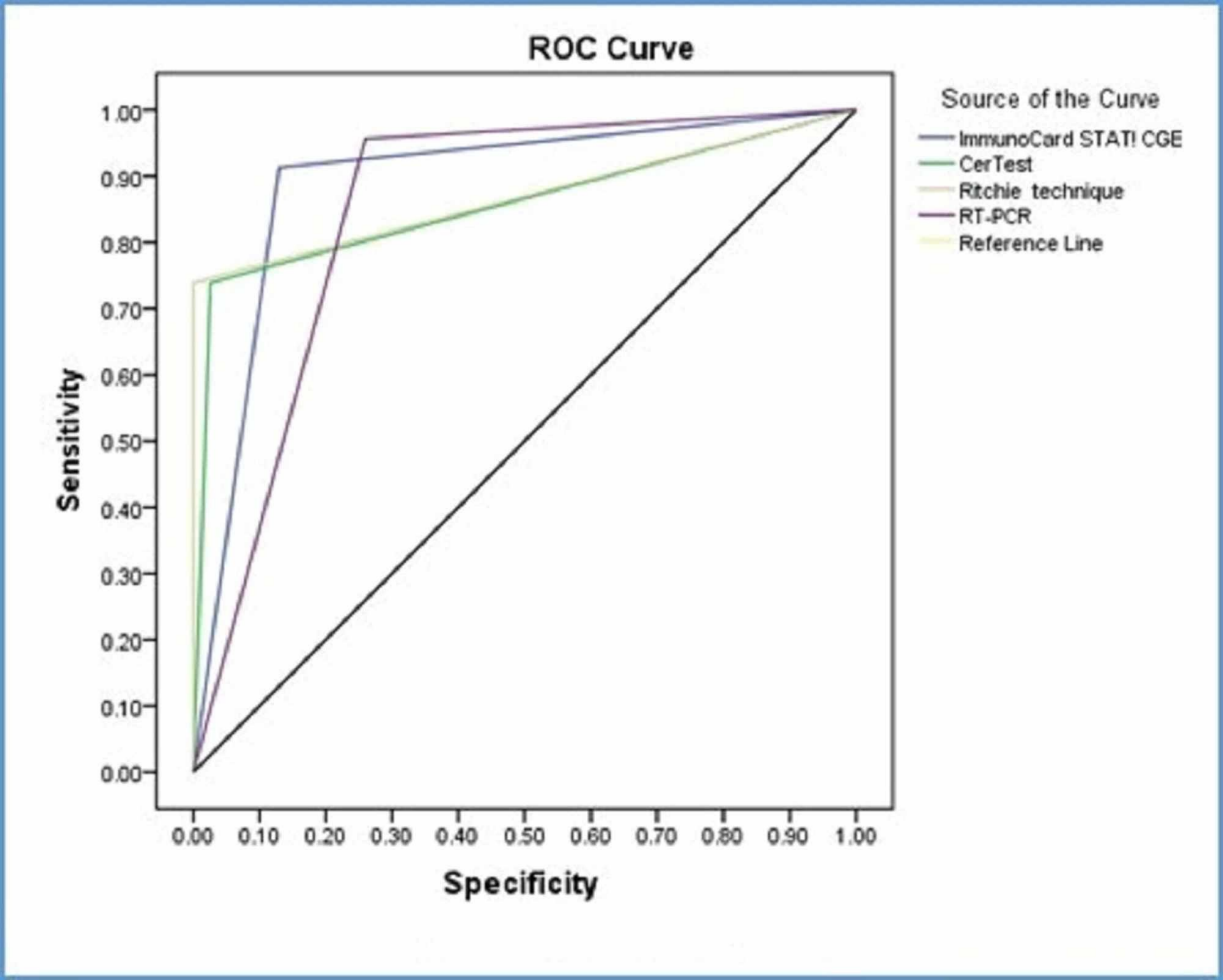
Sensitivity, specificity, and accuracy of diagnosis tools with direct smears

When direct smears assumed as a nominated golden standard, real-time PCR showed the highest sensitivity followed by ImmunoCard STAT! CGE and then the CerTest and Ritchie technique (Table 3). ImmunoCard STAT! CGE was excellent. The area under the curve is 0.892, Std. Error=0.041, p-value <0.0005 with 95% CI (0.812-0.972). CerTest was excellent. The area under the curve is 0.857, Std. Error=0.056, p-value<0.0005 with 95% CI (0.746-0.967). The Ritchie technique was excellent. The area under the curve is 0.870, Std. Error=0.056, p-value<0.0005 with 95% CI (0.760-0.979). Real-time PCR was excellent. The area under the curve is 0.848, Std. Error=0.041, p-value<0.0005 with 95% CI (0.768-0.929) (Figure 2).

**Table 3:** Sensitivity, specificity, and accuracy of diagnostic tools with direct smears N: Negative, P: Positive, TP: True-positive, FP: False-negative, TN: True-negative, FN: False-negative, PPV: Positive predictive value, NPV: Negative predictive value ImmunoCard STAT!: Meridian Bioscience, Germany; CerTest: Biotec, Zaragoza, Spain

Test		Direct smears		Sensitivity %	Specificity %	PPV %	NPV %	95% CI	P value
		P	N						
Immuno Card STAT! CGE	P	^TP^21	^FP^10	(91.3)	(87.01)	(67.74)	(97.10)	(0.81-0.98)	P < 0.001
	N	^FN^2	^TN^67						
CerTest	P	^TP^17	^FP^2	(73.91)	(97.40)	(89.47)	(92.59)	(0.75-0.97)	P < 0.001
	N	^FN^6	^TN^75						
Ritchie	P	^TP^17	^FP^0	(73.91)	(100)	(100)	(92.77)	(0.76-0.98)	P < 0.001
	N	^FN^6	^TN^77						
Real-time PCR	P	^TP^22	^FP^20	(95.65)	(74.03)	(52.38)	(98.28)	(0.77-0.93)	P < 0.001
	N	^FN^1	^TN^57						

**Figure 3:**
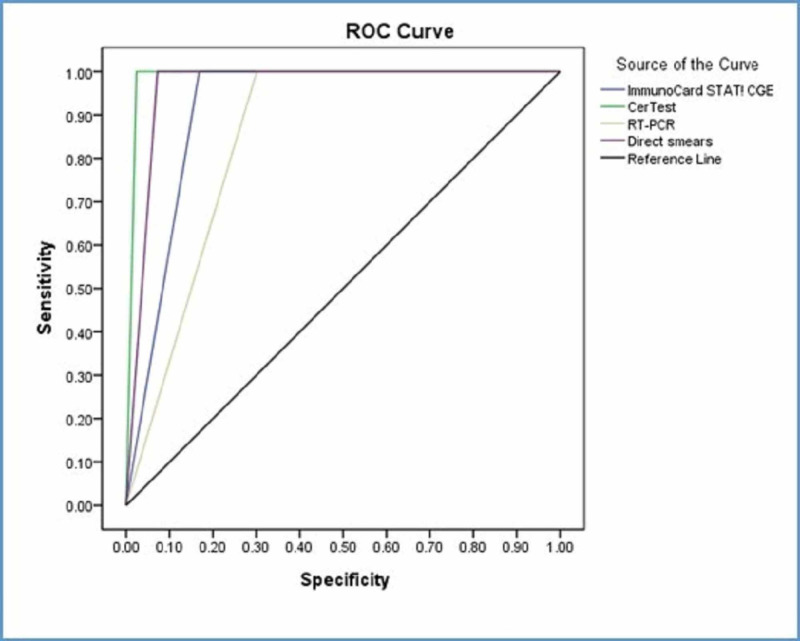
Sensitivity, specificity, and accuracy of diagnostic tools with the Ritchie technique

When the Ritchie technique considered as a nominated golden standard, all tests had (100%) sensitivity with the Ritchie technique (Table 4). ImmunoCard STAT! CGE was outstanding. The area under the curve is 0.916, Std. Error=0.027, p-value<0.0005 with 95% CI (0.862-0.969). CerTest was outstanding. The area under the curve is 0.988, Std. Error=0.010, p-value<0.0005 with 95% CI (0.969-1). Real-time PCR was excellent. The area under the curve is 0.849, Std. Error=0.038, p-value<0.0005 with 95% CI (0.776-0.923). Direct smears were outstanding. The area under the curve is 0.964, Std. Error=0.018, p-value<0.0005 with 95% CI (0.929-0.998) (Figure 3).

**Table 4:** Sensitivity, specificity, and accuracy of diagnostic tools with the Ritchie technique N: Negative, P: Positive, TP: True-positive, FP: False-negative, TN: True-negative, FN: False-negative, PPV: Positive predictive value, NPV: Negative predictive value ImmunoCard STAT!: Meridian Bioscience, Germany; CerTest: Biotec, Zaragoza, Spain

Test		Ritchie		Sensitivity %	Specificity %	PPV %	NPV %	95% CI	P-value
		P	N						
Immuno Card STAT! CGE	P	^TP^17	^FP^14	(100)	(83.13)	(54.84)	(100)	(0.86-0.97)	P < 0.001
	N	^FN^0	^TN^69						
CerTest	P	^TP^17	^FP^2	(100)	(97.59)	(89.47)	(100)	(0.97-1.00)	P < 0.001
	N	^FN^0	^TN^81						
Real-time PCR	P	^TP^17	^FP^25	(100)	(69.88)	(40.48)	(100)	(0.78-0.92)	P < 0.001
	N	^FN^0	^TN^58						
Direct smears	P	^TP^17	^FP^6	(100)	(92.77)	(73.91)	(100)	(0.93-1.00)	P < 0.001
	N	^FN^0	^TN^77						

## Discussion

Intestinal parasitic infections are among the major public health problems in tropical and sub-tropical parts of the world, particularly in rural communities with poor sanitation and poor personal hygiene behaviors. The goal of our study was to compare the sensitivity and specificity of three diagnostic methods: light microscopy, ICTs, and real-time PCR for the detection of *G. lamblia* in clinical human fecal samples.

Light microscopy is still considered the reference standard access to the fecal parasitological laboratories but there is growing interest in alternative detection methods to overcome the current limitations of microscopic examination. We accomplished a microscopic examination by using direct smears, and the Ritchie technique. The Para-Pak trichrome stain was used to confirm the morphological features for trophozoites and cysts for protozoa parasites. Meanwhile, DNA detection by PCR is offering a better turnaround time and ICTs are commercially available and widely used in clinical laboratories, with limited capacities for diagnostic complexity [16].

Stool samples were collected from non-Saudi workers applying for a residence permit (Iqama). Non-Saudis represent around 30% of the population in Saudi Arabia [17]. All subjects submitted non-formed stool specimens ranging from soft to watery. Similar results were revealed by a study about the detection of gastrointestinal pathogens in migrant workers in Qatar [18].

Out of the 100 fecal samples analyzed for *G. lamblia* detection, 42 were found positive by real-time PCR, 31 by ImmunoCard STAT! CGE, 23 by direct smears, 19 by CerTest, and 17 by the Ritchie technique. In 2016, Ahmed and others [19] studied the prevalence of three recognized enteric protozoa; *E. histolytica*, *G. lamblia*, and *Cryptosporidium* spp. among children in Bangladesh. Regarding the variation in their study subject, the prevalence of giardiasis was (50.6%), (26.7%), and (3.3%) by using real-time PCR, ICTs, and direct microscopy respectively. The authors also observed that the positive samples by real-time PCR but negative by the other techniques showed higher Ct values due to the small number of parasites in those samples that fell below the other methods limit of detection. Our finding is in agreement with that observation. The variation in our results between microscopic examination using direct smears and the Ritchie technique is due to the ability of each technique for the detection of cyst and trophozoite. Direct smears detect both cyst and trophozoite stages while the Ritchie technique is applicable to the cyst stage only [10]. Using direct smears, we observed two cases with only trophozoites and one case with both trophozoites and cysts while none of the trophozoites was detected with the Ritchie technique.

A study conducted in Mozambique for the detection of intestinal parasites in fecal samples showed that the sensitivity of real-time PCR for the detection of each of the parasite species was higher than direct smears, Ritchie technique, Kato smear, Baermann, and coproculture [20]. The workers noticed that real-time PCR-positive samples, but negative during microscopy had higher Ct values (significantly lower DNA loads) than samples that were positive by both techniques. On the other hand, some samples were positive only by microscopy but negative by real-time PCR. Moreover, a recent study reported the detection of a low number of parasites by light microscopy but negative by PCR [21]. In our study, one sample was positive by routine microscopy and undetectable by real-time PCR; this was fairly in agreement with the above-mentioned studies. The microscopic examination and the DNA isolation usually use a small portion of feces so it might be in some instances that the parasite is observed by light microscopy but missed by PCR. This variation between techniques might be due to the dispersion of cysts within the subsampling due to the nonhomogeneous structure in some stool samples besides the reported non-uniform structure of the intestinal parasites' excretion in the stool [22].

Nominating real-time PCR as a gold standard, ImmunoCard STAT! CGE showed a higher sensitivity (59.5%) than both direct smears and the Ritchie technique (52.4% and 40.5%), respectively (Table 2). A study in Egypt revealed higher sensitivity for ICTs than microscopy when comparing the PCR technique detecting the 18S and tpi genes of giardiasis [23]. Rapid immunoassays kits provide sufficient sensitivities and specificities in direct detection, particularly when using a single stool sample [24].

Seven negative samples in real-time PCR were positive by other tests, therefore, they were suspected to be false-positive; six with ImmunoCard STAT! CGE and one with CerTest. The one giardiasis positive sample with CerTest could be due to cross-reactivity, as the specimen comprised other intestinal parasites, including *E. histolytica*. Interestingly, this sample was negative by direct smears as well. However, the suspected six false-positive samples in ImmunoCard STAT! CGE could be due to a cryptic giardiasis infection or the persistence of antigen shading after treatment rather than true cross-reactivity [25]. *G. lamblia* DNA can be found in feces after the parasite clearance and may occasionally produce false-positive PCR results. Moreover, real-time PCR and ICTs may generate false-negative results because of the intermittent patterns of cysts shedding and the number of stool samples required for analysis [26].

Using direct smears as the reference method, real-time PCR showed the highest sensitivity, followed by ImmunoCard STAT! CGE, then CerTest, and finally the Ritchie technique (95.7%, 91.3%, 73.9%, and 73.9%), respectively (Table 3). This finding contrasts with a study in Germany, as the two ICTs showed a sensitivity of 72.9% and 93.8%, respectively, while PCR showed a sensitivity of 85.4% [27]. This could be explained by the high concentration of DNase in the samples that inhibit the PCR diagnostics from this material.

Using the Ritchie technique as the reference method, the sensitivity for all the diagnostic tests has reached 100% while the specificities ranged from 69.9% to 97.6% (Table 4). Another study reported the low sensitivity of ImmunoCard STAT! CGE (65.3%) by using formol concentration as the golden standard for the detection of *Giardia* in fecal samples in Central Africa [28]. The low sensitivity of ICTs could be related to the non-specificity of the antibody-based method owing to cross-reactivity with other microorganisms. Meanwhile, a higher sensitivity of the ImmunoCard STAT! CGE (93.5%) was observed in a previous study [29]. A similar observation was reported after using saturated sodium nitrate ﬂotation as the golden standard for the detection of *G. lamblia* [30], and the sensitivities of the PCR and the immunoﬂuorescence assay were 97.3% and 91.9%, respectively, while the speciﬁcity of both was 100%.

## Conclusions

In conclusion, with some variations, all the investigated techniques were applicable for the detection of *G. lamblia* in stool samples. The real-time PCR assay was revealed as the most accurate diagnosis of giardiasis. ICTs proved to be practical, easy to perform, time-saving, and beneficial to analyze a low number of samples. It can be a valuable diagnostic tool in case microscopic expertise is limited. Wet mount microscopy and the Ritchie technique are still important methods for the detection of gastrointestinal parasites when a skilled technologist tests the samples.
